# Antiviral and Immunomodulation Effects of Artemisia

**DOI:** 10.3390/medicina57030217

**Published:** 2021-02-27

**Authors:** Suhas G. Kshirsagar, Rammohan V. Rao

**Affiliations:** 1 College of Ayurveda, Mount Madonna Institute, 445 Summit Road, Watsonville, CA 95076, USA; 2 California College of Ayurveda, 700 Zion Street, Nevada City, CA 95959, USA

**Keywords:** Artemisia, Artemisinin, ARTs, phytochemicals, SARS-CoV-2

## Abstract

*Background and Objectives:* Artemisia is one of the most widely distributed genera of the family Astraceae with more than 500 diverse species growing mainly in the temperate zones of Europe, Asia and North America. The plant is used in Chinese and Ayurvedic systems of medicine for its antiviral, antifungal, antimicrobial, insecticidal, hepatoprotective and neuroprotective properties. Research based studies point to Artemisia’s role in addressing an entire gamut of physiological imbalances through a unique combination of pharmacological actions. Terpenoids, flavonoids, coumarins, caffeoylquinic acids, sterols and acetylenes are some of the major phytochemicals of the genus. Notable among the phytochemicals is artemisinin and its derivatives (ARTs) that represent a new class of recommended drugs due to the emergence of bacteria and parasites that are resistant to quinoline drugs. This manuscript aims to systematically review recent studies that have investigated artemisinin and its derivatives not only for their potent antiviral actions but also their utility against the severe acute respiratory syndrome coronavirus 2 (SARS-CoV-2). *Materials and Methods*: PubMed Central, Scopus and Google scholar databases of published articles were collected and abstracts were reviewed for relevance to the subject matter. *Conclusions*: The unprecedented impact that artemisinin had on public health and drug discovery research led the Nobel Committee to award the Nobel Prize in Physiology or Medicine in 2015 to the discoverers of artemisinin. Thus, it is clear that Artemisia’s importance in indigenous medicinal systems and drug discovery systems holds great potential for further investigation into its biological activities, especially its role in viral infection and inflammation.

## 1. Introduction

Medicinal plants, which are undervalued, have an important place in modern medicine owing to the multitude of active principles that nature provided through millions of years of evolution. These numerous plant chemicals or phytochemicals possess far reaching, biologically active, beneficial effects and provide protection to the plants from insects, bacteria, virus and other predators. These phytochemicals either alone or in combination affect multiple pathways simultaneously to produce the desired pharmacological effect. Many medicinal plants or herbs are revered by the ancient medical traditions (Chinese medicine, Ayurveda, Native Americans, etc.) due to their healing benefits and about 40% of modern medicines are derived from plants [1,2,3]. The development of antibacterial and anti-infectious agents is a major focus in modern medical research. Plant-based antiviral formulations have been studied for their therapeutic potential in the management of various viral diseases including influenza, human immunodeficiency virus (HIV), herpes simplex virus (HSV), hepatitis, and coxsackievirus infections [4,5,6,7]. 

One particular plant that has garnered a lot of attention in lieu of the COVID-19 epidemic is Artemisia which is one of the largest and most widely distributed genera of the family Astraceae (Compositae) [6,8]. Artemisia is a varied genus consisting of more than 500 diverse species and is found in the temperate zones of Europe, Asia and North America [9,10,11]. Evidence-based in vitro and in vivo studies on several species of Artemisia resulted in the identification of numerous phytochemicals with varied pharmacological activities, including terpenoids, flavonoids, coumarins, caffeoylquinic acids and sterols [12,13]. The first clinical trial of Artemisia extract in human patients with malaria was conducted in August 1972. Following that trial, the active compound in the Artemisia extract was isolated and identified as artemisinin. Several derivatives or synthetic compounds with key structures similar to artemisinin have now been developed including artesunate and piperaquine from *A. annua* and piperitone and trans-ethyl cinnamate from *A. judaica* that have potent antiviral and anti-inflammatory activities [9,14,15,16]. A combination of artemisinin and its derivatives (ARTs) is now recommended by the World Health Organization (WHO) for the treatment of malaria. 

## 2. Methods

For the review, we conducted a search of PubMed Central, Scopus and Google scholar databases of articles published from 2000 to 2020 to include the most recent literature. The search was limited to articles in English and abstracts were reviewed for relevance to the subject matter of pharmacological perspective on Artemisia. Articles containing combinations of MeSH terms ‘Artemisia’ ‘artemisinin’, ‘artesunate’, ‘phytochemistry’, ‘diseases or conditions’, ‘in vitro or in vivo experiments’, ‘COVID-19′, ‘SARS-CoV-2′ and ‘mechanistic actions’ were collected. Boolean terms (AND/OR/NOT) were added to combine or exclude keywords in the search resulting in more focused literature. 

## 3. Ethnopharmacology

The isolation and identification of potent compounds from the genus Artemisia, particularly artemisinin and its derivatives using novel drug discovery methods, prompted the Nobel Committee to award the Nobel Prize in Physiology or Medicine in 2015 for its impact on public health [17,18]. This spurred the interest of several researchers to study the phytochemical and pharmacological properties of other species of the genus Artemisia. 

Nearly 45 different species of Artemisia grow in India and in the Indian subcontinent and is mainly used as a medicinal plant [8,19]. Ayurveda describes two species *A*. *absinthium* and *A. maritima,* popularly known as Mugwort that vary slightly in their qualities and actions as shown in Table 1. 

In the Ayurvedic system of medicine, the term ‘*prabhava*’ refers to the ‘instinct intelligence’ of a plant in eliciting a wide range of medicinal effects [21,22]. *A*. *absinthium* and *A. maritima* are revered, owing to their *prabhava* and are recommended in Ayurveda for infections, inflammation, skin and liver diseases, respiratory conditions, neurological conditions and as an insecticidal (*krimighna*) [6,8]. 

The pharmacological actions and properties of the various Artemisia species from several geographic locations are listed in Table 2. Basically, the plant has been used as an anti-malarial, anti-spasmodic, anti-inflammatory, febrifuge, cardiac stimulant, anthelmintic, headaches, dyspepsia, liver and kidney tonic, to improve memory, for digestive and respiratory issues and as a hypertensive and anticoagulant.

The wide variety of actions stems from the fact that these various species of Artemisia possess high content of alkaloids, lactones, flavonoids, phenols, quinines, tannins and terpenoids all of which play a role in the growth of the plant or provide protection from pathogens or predators [6,9,44,45]. 

## 4. In Vitro and In Vivo Studies

We review some of the recent in vivo and in vitro studies of various extracts and formulations of Artemisia. The research studies utilized aqueous, methanol, chloroform or acetone extracts, essential oils or oil based extracts or dried powders of various species of Artemisia. The studies were performed on bacterial, viral or fungal cultures, cultured cells or animal models with limited studies on humans. 

In the light of the COVID-19 pandemic, some species of Artemisia including but not limited to *A. annua*, *A. absinthium*, *A. vulgaris*, *A. maritima* and *A. indhana* are receiving greater attention from researchers as they hold great potential for their powerful anti-infectious, antiviral and anti-inflammatory activities [6,12,46,47,48]. Recent studies are now pointing to the exciting roles of artemisinin and its derivatives (ARTs) as potential drug candidates against SARS-CoV-2 owing to their potent antiviral and anti-inflammatory properties. 

### 4.1. Anti-Carcinogenic Activity

Various species of the Artemisia plant have been shown to suppress the growth of numerous cancer cell lines including leukemia, colon cancer, renal cell carcinoma and breast cancer cells [28,49,50]. Phytochemical analysis of the various extracts revealed the presence of coumarins, flavonoids, anthocyanins, cardiac glycosides and tannins. These phytochemicals and their derivatives exhibit growth inhibitory properties through multiple actions including blocking angiogenesis, triggering apoptosis or cell cycle arrest and disrupting cell migration [51,52,53]. Researchers are now focusing their efforts on ARTs that appear to be broad-spectrum antitumor agents based on their efficacy and safety [54,55]. 

In a randomized, double-blind, placebo-controlled pilot trial involving 23 subjects, the anticancer effect and tolerability of oral artesunate in colorectal cancer (CRC) was determined. The primary outcome measure was the proportion of tumor cells undergoing apoptosis. Despite the fact that it was a small study size with variability in quantitating immunohistochemical markers, the results clearly indicated selective cytotoxicity of oral artesunate. 

In addition to the above mentioned study, other clinical trials involving patients with solid tumors including colorectal carcinoma, breast cancer, hepatocellular carcinoma and lung cancer have been completed with encouraging results. In all these studies, ARTs inhibited growth of solid tumors with no evident toxicity and with a low incidence of adverse effects thus highlighting their role as promising anti-cancer agents [54,56,57].

### 4.2. Anti-Oxidant Activity

The phytochemicals and their derivatives, extracts and essential oils derived from the Artemisia plant have a unique property of being reactive oxygen species (ROS) modulators. In some cases they exhibit strong antioxidant and radical scavenging activity against hydroxyl ion and hydrogen peroxide and display excellent protective effect by strengthening the antioxidant defense system and lowering the generation of ROS [6,58].

In other situations, especially involving cancer cells, ARTs triggered ROS production leading to mitochondrial dysfunction and autophagy of leukemia cell lines. ARTs-induced ROS production triggered apoptosis in various tumor cell lines studies, including neuroblastoma, glioblastoma, T-cell lymphoma and breast cancer cells [54]. In studies using mouse models of cancer, ARTs induced ROS production leading to the inhibition of growth of ovarian cancer [54]. 

The mechanism of action of ARTs involves binding to ferrous iron (e.g., heme) and triggering the generation of ROS, which results in cytostatic or cytotoxic effects. The production of ROS can also trigger cellular damage through the peroxidation of membrane lipids, activation of pro-apoptotic pathways or creating genomic and mitochondrial DNA instability [59]. Thus, the ROS modulating properties exhibited by the various phytochemicals isolated from different species of Artemisia highlight the importance of exploring the therapeutic uses of these compounds in pathological conditions that feature oxidative stress. 

### 4.3. Anti-Bacterial and Anti-Parasitic Activity

The plant extracts and compounds obtained from Artemisia species have been shown to be powerful inhibitors of bacteria and parasites [9]. Mechanistic studies demonstrate the bactericidal properties of some of these phytochemicals against Gram-negative or Gram-positive bacteria involving the destruction of the bacterial membrane [6,28,60,61]. Notable among the phytochemicals is ARTs that represent a new class of antibacterial drugs [9,14,15].

ARTs also possess potent antimalarial properties and are effective against both asexual and sexual parasite stages. In several clinical trials involving both ARTs and quinine, ARTs outperformed quinine in terms of mean parasite clearance time, fever clearance time, coma resolution times and incidence of adverse effects [14,15,16,62]. Artemisinin-based therapies are now recommended due to the resistance displayed by bacteria and parasites to quinoline drugs.

### 4.4. Anti-Fibrotic Effects

In addition to the above mentioned pharmacological properties, ARTs are also known for their anti-fibrotic effects [29,63,64]. The role of ARTs in blocking the development or progression of fibrotic phenotypes has been studied in animal models of pulmonary fibrosis, renal fibrosis, hepatic fibrosis, and other types of tissue fibrosis suggesting the potential utility of these compounds as anti-fibrotic agents. The effects of ARTs against profibrotic processes include induction of apoptosis, inhibition of proliferation, blocking differentiation of tissue-specific myofibroblast precursors or preventing the accumulation of tissue myofibroblasts that provoke tissue fibrosis [6,63]. In addition, ARTs block the expression of extracellular matrix (ECM) genes and pro-fibrotic genes in myofibroblasts thereby antagonizing cellular processes that promote accumulation of fibrotic tissue. ARTs also inhibit angiogenesis either through direct effects on endothelial cells or indirectly by downregulating pro-angiogenic gene expression in angiogenesis-supporting non-endothelial cells. With its anti-fibrotic role in disease models across several species and multiple tissues involving diverse mechanisms, artemisinin-based therapeutics for treatment of fibrotic diseases may prove efficacious in humans [64].

### 4.5. Role in Neurodegeneration

Extracts of several Artemisia species exhibit neuroprotective effects against focal ischemia-reperfusion-induced cerebral injury, microglial cytotoxicity and glutamate excitotoxicity [65]. Furthermore, Artemisia protects neurons against mitochondrial potential loss, attenuates reactive oxygen species and protects neurons against H_2_O_2_-induced death by upregulating the Nrf2 pathway [66]. ARTs improve learning and memory in mouse models of Alzheimer’s disease mice by blocking Aβ25-35-induced increase in the levels of inflammatory cytokines IL-1β, IL-6 and TNF-α and by restoring the autophagic flux and promoting the clearance of Aβ fibrils [67,68]. 

Recently, three different subtypes of Alzheimer’s disease (AD) have been described [69]. The type-3 AD classified as infectious or Krimi (ayurveda classification of AD) is the result of exposure to virus or biotoxins, such as mycotoxins, and features chronic inflammation [69,70]. Owing to their powerful antiviral and anti-inflammatory properties, ARTs may serve as excellent drug candidates for type-3 AD. 

### 4.6. Anti-Inflammatory Activity

Artemisia species exhibit powerful anti-inflammatory effects. Several sesquiterpenes derived from Artemisia and their derivatives including artemisinin, artesunate, dihydroarteannuin, artemisolide, eupatilin, scoparone, capillarisin and scopoletin have received special attention due to their role in blocking inflammation. Using animal models, ARTs were found to be effective in treating inflammatory conditions including rheumatoid arthritis, systemic lupus erythematosus, multiple sclerosis and allergic disorders [71]. 

Some of the anti-inflammatory mechanisms include: (1) inhibition of the iNOS and COX-2 pathways; (2) suppression of ERK and NF-κB signaling; (3) inhibition of pathogenic T cell activation; (4) suppressing B cells activation and antibody production; and (5) inhibition of Akt phosphorylation and IκB degradation through the PI3K/Akt signaling pathway downstream of TNF-α [72,73,74,75]. Thus, the varied mechanisms through which these phytochemicals derived from Artemisia exhibit their anti-inflammatory effects warrant investigation into their role as therapeutic candidates for inflammatory conditions and autoimmune disorders.

### 4.7. Anti-Viral

Several phytochemicals isolated from various Artemisia species exhibit significant antiviral activity [76]. ARTs have turned out to be the most promising antiviral drug candidates with activities against hepatitis B and C viruses, human herpes viruses HSV-1 and HSV-2, HIV-1 and influenza virus A in the low micromolar range [77,78,79,80,81,82]. In most cases, ARTs inhibited the central regulatory processes of viral-infected cells (NF-κB or Sp1-dependent pathways), thus blocking the host-cell–type and metabolic requirements for viral replication [80]. 

Owing to their potent anti-inflammatory, immunoregulatory and antiviral properties, ARTs are being pursued for their activity against SARS-CoV-2 infection. Researchers used in silico approaches to investigate if artemisinin or its derivatives could physically bind any of the COVID-19 target proteins including SARS-CoV-2 spike glycoprotein, spike ectodomain structural protein, the main protease of the virus (M^Pro^) or spike receptor-binding domain, thereby preventing SARS-CoV-2 from binding to the host receptor ACE2 [83,84,85,86,87,88,89]. ADMET (absorption, distribution, metabolism, excretion and toxicity) analysis of artemisinin showed that it was non-cytotoxic, had good aqueous solubility and a good permeability through the blood–brain barrier with a promising therapeutic potential. Furthermore, molecular docking studies revealed that artemisinin bound to all four proteins and in some cases displayed better binding modes than hydroxychloroquine [85,86,87,88,89]. Thus, ARTs could serve as best leads for further drug development process for SARS-CoV-2 infection.

Several investigators have now shown that extracts from different species of Artemisia are active against SARS-CoV-2 [79,86,90,91]. Results from recent studies indicate that ARTs impair SARS-CoV-2 viral infection by modulating several host cell metabolic pathways thus making them attractive candidates for COVID-19 [85,86,92]. The mechanism of antiviral activity may be through the induction of cellular ROS, blunting the PI3K/Akt/p70S6K signaling pathway, binding to NF-κB/Sp1 or inducing a endocytosis inhibition mechanism, all of which lead to inhibition of viral replication and growth [85,93,94]. The above mentioned results have spurred the interest of few groups to embark on clinical trials to evaluate the safety and efficacy of ARTs in the treatment of subjects with SARS-CoV-2 viral infection.

In a recently published controlled clinical trial, 41 patients with confirmed COVID-19 were divided into two groups. While 18 subjects served as the control group, the experimental group (*n* = 23) received a combination of artemisinin-piperaquine (AP). AP was orally administrated with a loading dose of two tablets (artemisinin 125 mg and piperaquine 750 mg) on the first day, followed by a low dose of one tablet/day (artemisinin 62.5 mg and piperaquine 375 mg) for six days [95]. The primary outcome was the percentage of participants with undetectable SARS-CoV-2 on days 7, 10, 14, and 28 following the treatment. The results indicated that: (1) the average time to achieve undetectable SARS-CoV-2 RNA in the AP group was significantly less than that in the control group; (2) the elimination rate of SARS-CoV-2 RNA in the AP group was significantly higher than that in the control group; and (3) the length of hospital stay for the AP group was significantly lower than that in the control group. Although the study had insufficient sample size and trial design, nevertheless, the safe toxicity profile and immunoregulatory activities makes AP an excellent drug candidate against SARS-CoV-2 infection [95].

Transforming Growth Factor-beta (TGF-β) plays an important role in modulating the immune system and displays different activities on different types of immune cells. SARS-CoV-2 infection is accompanied by a cytokine storm together with edema and pulmonary fibrosis at the end stage of the infection. SARS-CoV-2 also up-regulates TGF-β expression which may partly explain the cytokine storm and fibrosis in the lung [94,96,97]. Efforts are underway to discover novel and specific small molecules that can potently block TGF-β expression with negligible side-effects. Artemisinin and its derivatives have been shown to be suppressors of TGF-β in several models of inflammatory diseases [64,98,99,100]. A randomized, open-label Phase IV study is underway to evaluate the safety and efficacy of a proprietary formulation of ARTs in adult COVID-19 patients with symptomatic mild-moderate COVID-19 [101]. In addition to its potent antiviral activity, the drug is expected to mitigate the TGF-β mediated inflammatory injury associated with the cytokine storm and viral sepsis in these patients. Initial results show that the ARTs-based drug has a very favorable safety profile and significantly accelerated the recovery of patients with mild-moderate COVID-19 infection [101]. Thus inhibition of TGF-β signaling by ARTs may be an attractive therapeutic strategy making them excellent drug candidates against SARS-CoV-2 infection.

## 5. Conclusions and Future Direction

Several phytochemical derivatives and lead molecules have been developed from medicinal plants for various significant therapeutic activities [4,5,94,102,103]. Scientists routinely investigate medicinal plants just for that one single and potent compound responsible for the therapeutic effect [104]. Studies comparing the action of whole plant extracts to the action of purified preparation show that, in many cases, the potency of the purified preparation declines at each step of fractionation [105]. Since the therapeutic effect may be the result of the combination of several compounds present in the medicinal plant, a complex mixture of compounds has a greater effect than isolated compounds [106]. The advantages of a combinatorial approach may be the synergy exhibited by the various components, enhanced bioavailability, cumulative effects and affecting an entire network of pathways in tandem [107]. 

Artemisia has prominence in Chinese and Ayurvedic medicinal systems for its numerous therapeutic properties. Among the phytochemicals present in the plant, the lactone derivative artemisinin and its derivatives—termed ARTs—are very promising owing to their multiple pharmacological actions [4,7,85,93,94]. Recent studies point to ARTs as attractive candidates for SARS-CoV-2 and they are a major focus in medical research [4,85,92,103]. SARS-CoV-2 infection manifests as a mild respiratory tract infection and influenza-like illness to a severe disease with accompanying lung injury (in severe cases lung fibrosis), oxidative stress, multisystem inflammatory conditions, multi-organ failure and neurological issues [108,109,110,111]. The role of ARTs as an antioxidant and anti-inflammatory and to be able to block tissues fibrosis together with its safety and low toxicity profile makes it an excellent drug candidate against SARS-CoV-2 infection [85,94].

## Figures and Tables

**Table 1:** Ayurveda describes two species *A. absinthium* and *A*. *maritima* that vary slightly in their qualities and actions. Both plants popularly known as Mugwort are revered in Ayurveda for their anti-infectious and insecticidal (*krimighna*) properties [20].

	*A. absinthium*	*A. maritima*
Names	Mugwort, Indian wormseed, Damanaka, Davana, Dona, Douna, Davanamu	Old woman, Mugwort, worm seed, Kirmani, Chuhara, Dirmana
Qualities (*guna*)	Light (laghu), Dry (ruksha), Hot (teekshana)	Light (laghu), Dry (ruksha), Hot (teekshana)
Taste (*rasa*)	Asringent (kashaya), Bitter (tikta)	Pungent (katu), Bitter (tikta)
Potency (*veerya*)	Hot (Ushna)	Hot (Ushna)
Post-digestive effect (*vipaka*)	Pungent (katu)	Pungent (katu)
Special Intrinsic Action (*prabhava*)	Insecticidal (*krimighna*), anti-pyretic (*jwaraghna*)	Insecticidal (*krimighna*), anti-pyretic (*jwaraghna*)
Uses	Optimizes kapha, pitta and vata (tridosha shamaka), anti-infectious, improves digestion, wound healing, respiratory and liver tonic	Optimizes kapha and vata (Kaphavata shamaka), anti-infectious, improves digestion, wound healing
Parts of plant used	Root, leaves, bark	Root, leaves, bark

**Table 2:** The pharmacological actions and properties of a subset of Artemisia species.

Species	Uses	Phytochemicals Isolated
*A.* *absinthium*	cardiac stimulant, anthelmintic, liver function, memory booster	Sesquiterpene lactones, polyphenolic compounds, flavonoids, tannins, lignins [23,24].
*A. abrotanum*	Insecticide, liver conditions	Flavonols, tannins, coumarins [6,8,25].
*A.* *afra*	coughs, colds, malaria, diabetes, bladder and kidney disorders	monoterpenoids, sesquiterpenes, glaucolides, guaianolides; flavonoids [23,26].
*A.* *annua*	Fever, malaria, fibrosis	Volatile oils, sesquiterpene lactones, phenolic compounds, flavones [23,27,28,29].
*A.* *asiatica*	cancer, inflammation, infections and ulcers	Volatile oils, flavones, alkaloids [6,30].
*A. arborescens*	Anti-inflammatory, Antihistaminic, Blood decongestant	Terpenes, flavone, fatty acids [6,31].
*A.* *douglasiana*	premenstrual syndrome and dysmenorrhea	Monoterpenes, sesquiterpene lactones [23,32]
*A.* *dracunculus*	antidiabetic and anticoagulant	Volatile oils, coumarins, polyphenolic compounds, glucoside [33,34].
*A.* *judaica*	Gastrointestinal disorders	Volatile components, phenolic compounds [6,35]
*A.* *maritima*	anthelmintic, liver function, GI issues	Volatile oils, fatty acids, polyphenolic compounds, sesquiterpene lactones [6,23]
*A.* *scoparia*	antibacterial, antiseptic, antipyretic	Volatile oils, fatty acids, coumarins, pyrogallol tannins, cholagogic components, flavonoids, flavones [6,36,37].
*A. t* *ripartite*	cold, sore throats, tonsillitis, headaches and wounds	Guaianolides, polysaccharides [6,38]
*A.* *verlotorum*	hypertension	Volatile oils, fatty acids [6,39,40].
*A.* *vestita*	inflammatory diseases	Volatile oils, flavonoids [41,42].
*A.* *vulgaris*	analgesic, anti-inflammatory, antispasmodic and liver disease	Terpenes, coumarins [6,43].

## Data Availability

Not Applicable.
